# Multidrug-resistant tuberculosis in KwaZulu-Natal, South Africa: An overview of patients’ reported knowledge and attitudes

**DOI:** 10.4102/phcfm.v8i1.1089

**Published:** 2016-06-17

**Authors:** Jayneetha Maharaj, Andrew Ross, Niren R. Maharaj, Laura Campbell

**Affiliations:** 1 Medical officer in MDR TB unit at King Dinuzulu Hospital, South Africa; 2 Discipline of Family medicine, University of KwaZulu-Natal, South Africa; 3 Obstetrics and Gynaecology, Prince Mshiyeni Memorial Hospital, South Africa; 4 Discipline of Family medicine, University of KwaZulu-Natal, South Africa

## Abstract

**Background:**

The incidence and prevalence of multidrug–resistant tuberculosis (MDR TB) in the province of KwaZulu-Natal, South Africa, are amongst the highest in the world. Previously, interventions have been largely biomedical based; however, there is growing opinion that interventions must include social aspects such as patient education and attitudes.

**Methods:**

This observational study assessed the knowledge and attitudes of 380 patients diagnosed with MDR TB at a centralised MDR TB unit in Durban. Data were collected using a questionnaire that was distributed to every third patient attending the outpatient MDR TB clinic. Data were collected over an 8-week period and analysed descriptively.

**Results:**

Just under half of the respondents had primary MDR TB. Most respondents were young, female unemployed and did not receive a social grant. Knowledge around diagnosis of MDR TB was generally adequate. There were important misconceptions about spread of the disease and duration of treatment. Most respondents received knowledge of MDR TB from healthcare workers. Some respondents received knowledge from friends, family and Sangomas and believed that the disease was caused by bewitchment or as a form of punishment.

**Discussion:**

The need for strengthening the role of primary care physicians in promoting education and providing support is highlighted. Further study is needed to investigate the high rate of primary MDR TB and to identify the unique challenges faced by women who have MDR TB. Future research could include the possibility of involving traditional healers in a contextually sensitive MDR TB education, training and support programme.

## Background

In 2012, it was estimated that worldwide, 3.7% of new and 20% of previously treated tuberculosis (TB) cases (totalling approximately 630 000) were classified as multidrug-resistant tuberculosis (MDR TB): that is, infection occurring with a *Mycobacterium tuberculosis* strain that is resistant to at least Rifampicin and Isoniazid (the two most effective anti-TB drugs).^1^ According to the World Health Organization (WHO) Global Tuberculosis Report for 2014, South Africa (SA) is amongst the 10 highest drug-resistant tuberculosis–burdened countries in the world when ranked by estimated burden of MDR TB,^2^ placed fourth behind only China, India and Russia.^2^ In the province of KwaZulu-Natal, there were 583 laboratory diagnosed cases of MDR TB in 2004, rising to 6630 in 2012, making the province the epicentre of SA’s MDR TB infection.^3^ There is thus a high and increasing burden of MDR TB disease in the province. Effective treatment of MDR TB is costly (approximately 50–200 times higher than treating drug-sensitive TB) and lengthy (treatment takes at least 18–24 months) and drug regimens are more complex to administer than those of drug-susceptible TB. These factors, and others, can lead to poor adherence resulting in treatment failure, particularly amongst HIV co-infected patients.^4^ Incomplete treatment of MDR TB patients is a serious situation, as at a patient level, it further amplifies drug resistance in *M. tuberculosis* strains and at a public health level may lead to the emergence of extensively drug-resistant TB.^4^ MDR TB is used as a marker of a TB control programme’s ability to adequately manage drug-susceptible TB, suggesting a serious problem in the TB control programme in SA.^2^

Given the complexity of management of MDR TB, a multifaceted approach is required, which includes swift, accurate diagnosis, availability of drugs and patients’ and healthcare professionals’ adequate knowledge and understanding of the disease. Reviewing associations between socio-economic factors, patients’ TB- and MDR TB-related knowledge, delayed diagnosis and poor treatment adherence are important as there is increasing consensus that socio-economic support and comprehensive patient education should be included in TB control interventions.^5^ Literature indicates that changes in socio-economic status, improving patients’ knowledge and attitudes strengthen TB control, and education interventions have recently been included in WHO TB control priorities.^5^ However, despite increasing interest in socio-economic aspects of TB and MDR TB, the importance of patients’ knowledge and attitudes towards the disease and treatment have been poorly researched.^6^

In SA, a hospital-based study carried out in 2011 reviewed healthcare professionals’ knowledge and attitudes towards TB and MDR TB. This study reported that knowledge around transmission and control were adequate,^7^ and although the health professionals were largely concerned about their own health, they generally held a positive attitude towards caring for patients with MDR TB.

A patient-based Ethiopian study in 2013 reported that a lack of knowledge and erroneous beliefs about TB and MDR TB were common amongst TB patients.^8^ An older study from 1999, based in China, illustrated that a lack of knowledge about TB and MDR TB resulted in stigmatisation, which resulted in underutilisation of services, delay in diagnosis and poor adherence to anti-TB medication.^9^ The study reported misconceptions and limited knowledge about TB, and its treatment amongst newly diagnosed TB patients and concluded that adequate counselling and education of patients may help to improve treatment adherence.^9^

In SA, there have been few studies on patients’ knowledge of and attitude to MDR TB, and hence, this study is important to guide primary care physicians around education and training of their patients and their families in an area of high MDR TB prevalence. The aim of the study was to explore the knowledge and attitudes of patients towards MDR TB in order to strengthen primary care practice and guide further research in this important area. Study questions were threefold: (1) What are the demographic profiles of patients with MDR TB? (2) What are their knowledge levels of aspects of MDR TB? (3) What are their attitudes towards MDR TB?

## Methods

The study design was descriptive and exploratory, and the study was conducted at the MDR TB Unit within a specialised TB Hospital in eThekwini, Kwazulu-Natal. Patients are referred to this unit from both private and public sector for initiation and follow-up consultations. Patients are diagnosed with MDR TB by GeneXpert testing, drug sensitivity testing or polymerase chain reaction–line probe assay, and only those patients with confirmed MDR TB are accepted for treatment at the unit. The MDR TB Unit in eThekwini is the largest specialised unit in KwaZulu-Natal, and on average, 250 new patients are initiated on MDR TB treatment each month. All MDR TB patients being followed up at the unit are seen monthly, with an average of 2500 patients seen monthly at this facility.

### Study participants and sampling

Study participants were patients in the outpatient department of the MDR TB Unit who were over the age of 18 years. A sample size calculation of 380 participants was derived with the assistance of a biostatistician using Creative Research Systems Survey Software^®^ (April 2015), incorporating statistical variables of a 95% confidence level, 5% significance interval, using a population size of 2500 patients. It was assumed that patients present for review at the clinic in a random order, and the researcher, who is based at the hospital, invited every third patient presenting in outpatient department to participate in the study until the sample size was reached. The study commenced on 23 July 2015, and the sample size was reached on 17 September 2015. Files of patients who had completed the questionnaire were marked to ensure that respondents did not participate on more than one occasion.

### Data collection instrument

The data collection instrument was a closed-ended questionnaire available in English and isiZulu in which participants were invited to respond to several predetermined potential responses. The questionnaire format comprised three sections: (1) demographic profile, (2) patients’ knowledge and (3) patients’ attitudes. The predetermined responses were selected based on a literature review of MDR TB patients’ knowledge and attitudes towards MDR TB^7,8,9^ and included responses around causes, symptoms, treatment and costs. The questionnaire was content validated with 10 patients to check their understanding of the questions and to correct any potential misconceptions. Patients who participated in the pilot study were not included in the data which were analysed for the study. Minor changes were made to the questionnaire following the pilot study. All questionnaires were anonymous and were not completed in the presence of the researcher so the potential for bias in responses was minimised. Nursing staff were available at the clinic to assist patients if necessary. All patients who were approached agreed to participate in the study.

### Statistical Analysis

Data entry was carried out by the researcher and data analysis performed using SPSS^®^ statistical package for descriptive statistics. Data were summarised using descriptive methods such as mean, frequency and range.

### Ethical considerations

Ethical approval was obtained from the Research Ethics Committee at University of KwaZulu-Natal (ethics number is BE 282/15). Institutional approval was obtained from the hospital management at the study site, and all patients signed informed consent before participating in the study.

## Results

A total of 380 patients with MDR TB completed the questionnaire. The results are presented in three sections: (1) demographic profile, (2) knowledge and (3) attitudes.

### Demographic profile

Most respondents were female (226/380; 59.5%). The largest age group was 31–40 years (143/368; 38.9%). Demographic variables are shown in Table 1.

**TABLE 1:** Demographic characteristics of participants.

Variable	Responses	%
**Age (years)**	368†	96.6
18–20	25	6.8
21–30	105	28.5
31–40	143	38.9
41–50	62	16.8
51–60	23	6.3
> 60	10	2.7
**Employed**	377†	99.2
Yes	87	23.1
No	290	76.9
**Education**	372†	97.8
No formal	14	3.8
Grade 1–7	54	14.5
Grade 8–12	240	64.5
Tertiary	64	17.2
**HIV status**	377†	99.2
Positive	299	79.3
Negative	65	17.3
Unknown	13	3.4
**On antiretroviral theraphy**	296†	99
Yes	279	94.3
No	17	5.7
**TB contact**	373†	98
** Yes**	298	80
No	75	20
**Previous TB**	366†	96.3
Yes	187	51
No	179	49
**Gender**	380	100
Male	154	40.5
Female	226	59.5
**Dwelling**	380	100
Formal	312	82.1
Informal	68	17.9
**Income (ZAR per month)**	170†	44.7
< R1000	55	32.4
1000–2999	58	34.1
3000–5999	22	12.9
6000–9999	12	7.6
10 000–15 000	11	6.5
> 15 000	11	6.5
**CD4 count**	298†	99.9
< 50	166	55.7
51–100	53	17.8
101–349	56	18.8
> 350	15	5.0
Unknown	8	2.7
**MDR TB† contact**	371	98
Yes	299	81
** No**	72	19

*Source*: Research study

MDR, multidrug–resistant; TB, tubercu-losis.

† Some data are missing as not all fields were completed by all the patients.

The vast majority (360/380; 94.7%) were from KwaZulu-Natal, and most reported that they lived in a formal dwelling (312/380; 82.1%). A majority reported that they were not currently formally employed (290/377; 76.9%), and most (113/170; 66.5%) reported a household income of less than R3000 per month. Less than half reported that they received a social welfare grant (154/374; 41.1%).

A minority (14/372; 3.8%) reported that they had no formal education, and few (64/372; 17.2%) had a tertiary education. Over three-quarters reported themselves to be HIV infected (299/377; 79%), and most of these reported they were taking anti-retroviral therapy (279/299; 93.3%). Just over half (187/366; 51%) had had previous TB. Most participants (299/371; 80.6%) reported having contact with someone who had MDR TB.

### MDR TB knowledge profile

Most (287/371; 77.3%) did not know the names of their MDR TB medications; however, a majority (271/361; 75.1%) reported that they knew what MDR TB was. Most (341/376; 90.1%) said that they received information about MDR TB from a healthcare worker. Others had received information on MDR TB from various sources including printed material (12/376; 3.2%), television (5/376; 1.3%), a friend/family (15/376; 4%) and a Sangoma (2/376; 0.5%). Most were correct in stating that MDR TB was caused by bacteria (278/372; 74.7%). When further questioned around causes of MDR TB, a quarter (99/372; 26.6%) stated that MDR TB was also caused/ spread by witchcraft. There was a non-statistical association between female gender and a belief that MDR TB could be spread by witchcraft (*p* = 0.051). Further details of patients’ knowledge can be seen in Table 2.

**TABLE 2:** Knowledge of MDR TB.

Variable	Responses	%
**Understand what MDR TB is**	361†	95
Yes	271	75
No	90	25
**Source Info**	376†	98.9
Health worker	341	90.7
Print media	12	3.2
TV	5	1.3
Family	9	2.4
Friend	6	1.6
Sangoma	2	0.5
**Do you about cough hygiene**	376†	99
Yes	140	37.2
No	236	62.8
**Duration of treatment, months**	376†	99
6	28	7.4
12	6	1.6
18	21	5.6
24	321	85.4
**Causes of MDR TB‡**	372†	97.9
** Don’t know**	130	34.9
TB bacteria	278	74.7
HIV	137	36.8
Not taking TB treatment properly	284	76.3
Witchcraft	99	26.6
**Spread of MDR TB‡**	357†	94
Coughing	336	94.1
Kissing	114	31.9
Touching	10	2.8
Sex	34	9.5
**Symptoms of MDR TB‡**	380	100
Loss of appetite	280	73.7
Loss of weight	292	76.8
Tiredness	260	68.4
Chest pain	275	72.3
**Knew names of MDR TB drugs**	371†	97.6
Yes	84	22.6
No	287	77.4

*Source*: Research study

MDR, multidrug–resistant; TB, tubercu-losis.

† Some data are missing as not all fields were completed by all the patients.

‡ Patients could indicate more than one option.

With regards to MDR TB transmission, most (336/357; 94.1%) believed the disease to be spread by coughing. A third (140/376; 37%) knew what was meant by cough hygiene, a third thought that MDR TB could be caused by kissing or touching (124/357; 34.7%) and less than 10% (34/357; 9.5%) believed that MDR TB could be spread by sexual intercourse.

Most (277/380; 72.8%) correctly identified symptoms associated with MDR TB, and the majority (306/373; 82.0%) knew how MDR TB was diagnosed. Three-quarters (284/372; 76.3%) recognised that poor adherence to TB medication was an important cause of MDR TB. All reported that drug treatment was ‘the best’ treatment for MDR TB with only 9/367 (2.5%) reporting that herbal medication would also be of use in treatment. Most replied that the duration of treatment for MDR TB was 24 months (321/376; 85.4%). However, some thought that treatment duration was 6 months (28/376; 7.4%), 12 months (6/376; 1.6%) and 18 months (21/376; 5.6%). Most wanted more information on MDR TB (359/377; 95.2%), and 223/233 (95.7%) would like information in a booklet form and 220/232 (94.8) indicated that a helpline or help desk would be helpful.

### MDR TB attitude profile

The majority (365/378; 96.6%) recognised that MDR TB was a major problem in SA. A few (30/272; 11%) believed they had acquired MDR TB because they were being punished by God. A small percentage of participants felt that there was an association between promiscuity and acquiring MDR TB (39/280; 13.9%). Only a small minority were aware of the cost of treatment for MDR TB (69/375; 18.4%), and a majority were of the opinion that defaulters should be held accountable, for example, using a fine (261/368; 70.9%). Some knew of a patient with MDR TB who was defaulting (58/365; 15.9%). Most (323/373; 86.6%) felt that they were receiving adequate support from their doctor and could communicate readily with their doctor. A small minority would stop their MDR TB medication when they felt better (23/376; 6.1%), and only (16/380; 4.2%) would stop their treatment if they felt worse. Details of patients’ attitudes towards MDR TB are presented in Table 3.

**TABLE 3:** Attitude towards MDR TB.

Variable	Responses	%
**Is MDR TB a major problem in South Africa**	378†	99.4
Yes	365	96.6
**Are you being punished by**	272†	71.6
God	30	11.0
Ancestors	7	2.6
Evil spirits	92	33.8
**Do you know the cost of MDR TB treatment**	375†	98.7
Yes	69	18.4
**Should defaulters be fined**	368†	96.8
Yes	261	70.9
**Do you take all medicines as prescribed**	373†	98.2
Yes	318	85.3
**Who is likely to contract MDR TB‡**	280†	73.7
Promiscuous	39	13.9
Criminals	10	3.6
Normal citizens	216	77.1
Homeless	35	12.5
Defaulters of TB treatment	234	83.6
MDR TB contacts	261	93.2
Those with HIV	153	54.6
**Know anyone who defaulted MDR TB treatment**	365†	96.1
Yes	58	15.9
**Did you receive appropriate care from your doctor**	373†	98.1
Yes	323	86.6
**Do you stop treatment if you are feeling better**	376†	98.9
Yes	23	6.1
**Do you stop treatment when you feel unwell**	380	100
Yes	16	4.2

*Source*: Research study

MDR, multidrug–resistant; TB, tubercu-losis.

† Some data are missing as not all fields were completed by all the patients.

‡ Patients could indicate more than one option.

## Discussion

Any study of MDR TB is important in KwaZulu-Natal as the province has one of the largest burdens of MDR TB worldwide, with an estimated prevalence of 30 MDR TB cases per 100 000 population.^10^ MDR TB treatment outcomes in KwaZulu-Natal are substantially worse than those published from other MDR TB cohorts.^10^

A total of 380 patients from the largest MDR TB treatment facility in the province participated in the study. The site offers a centralised treatment programme, and there are advantages to such a programme, as specialist doctors and nurses can attend to patients and ensure continuity of care. The sample contained more female than male respondents (60% versus 40%). There may be an associated co-morbidity of MDR TB with HIV infection, and in KwaZulu-Natal, HIV transmission rates from men to women have increased and poverty, the low status of women and gender-based violence have been cited as reasons for the disparity in HIV prevalence between men and women.^11^

The association between TB/MDR TB and HIV can be seen in the large percentage (79.3%; 299/377) of patients who were HIV positive. This is slightly higher than the 74.5% reported amongst patients with MDR TB at the same site in 2012.^12^ It is encouraging to note the increased uptake of highly active antiretroviral therapy (HAART) (85% in 2012 versus 93% in the study), suggesting that the new guidelines recommending initiation of anti-retroviral therapies in all HIV positive patients with MDR TB are being effectively implemented.^2^ However, despite the widespread availability of HIV testing and the roll out of the new treatment guidelines, the low CD4 count of less than 101 × 10^–6^ cells/L in 219 patients (219/298;73.5%) is of concern.

The high percentage of patients with previous TB (187/366; 51.1%) highlights the association between previous TB and acquiring MDR TB^2^ and reinforces the importance of correctly treating TB on the first occasion. However, of great concern was the 49% (179/366) of patients diagnosed with MDR TB who had no previous history of TB or previous exposure to anti-TB drugs. This is also much higher than the 8.9% (31/350) reported by Jacobs at this site in 2012.^12^ This rate of primary MDR TB, which is much higher than the 3.7% in the WHO figures,^1^ may be a reflection of a rising incidence of MDR TB in the community or better methods of detecting MDR TB through the use of GeneXpert and needs further study.

The literature recommends a need for ongoing research into multiple aspects of MDR TB, including social, economic and geographical factors.^6^ In support of such literature, the study explored social, economic and geographical contexts and found that a higher proportion of participants fell into the younger age groups, were unemployed, had relatively low income and were unlikely to have post-school training. Other literature reports that a higher level of schooling has a significantly positive effect on MDR TB knowledge and highlights the importance of addressing social issues in TB control programmes.^13^

Less than half of the participants received a disability pension, and most reported a household income of less than R 3000 per month, suggesting that many are extremely socially and economically vulnerable. It may be expensive for such a vulnerable patient to live in a city or travel from a distant home to a centralised MDR TB treatment site. Other MDR TB care models, such as community-based or home-based MDR TB care, may reduce patients’ financial burden. Data from this study support recommendations from other studies, which promote the establishment of decentralised MDR TB treatment programmes to reduce patients’ financial burdens in a high–MDR TB/HIV-prevalence setting such as KwaZulu-Natal, to improve patient adherence to medication.^14,15^ If MDR TB treatment becomes more decentralised, then the role of the Primary Care Physician in MDR TB education, training and support becomes invaluable.

Literature also describes the importance of patients’ knowledge of and attitudes to MDR TB in promoting adherence to MDR TB medication.^6^ The findings from the study illustrate that patients’ reported knowledge of MDR TB was good in that most respondents reported that they knew how MDR TB was spread. That few believed it to be caused by touching, kissing or sexual contact is in keeping with findings from other studies where respondents reported that MDR TB can be spread by means other than coughing including excessive alcohol consumption and smoking.^16^ Further exploration of patients’ knowledge around transmission may unearth other erroneous knowledge about MDR TB.

Most participants reported that they received information from a healthcare worker, and further study could focus on knowledge obtained from other sources such as television, family/friends and traditional healers. Any perceived association between source of knowledge and a belief that MDR TB could be caused by witchcraft or as a result of punishment from God would be useful. The perceived association between MDR TB and witchcraft found in the study has been also reported in studies carried out in Lesotho and Ghana.^17,18^ The Masai people of Kenya believe that MDR TB is a punishment.^19^ Such beliefs speak of a potential for educational intervention to extend beyond the biomedical, western-oriented education of the patient and family into the community using traditional healers. Traditional healers remain an important source of ‘healing’ and support for many patients, and their inclusion in MDR TB prevention and control may prove to be useful.

It was of concern that some had no knowledge of the names of the medication, and this finding concurs with another study in which patients with MDR TB did not know the names of their medications.^8^ Education around the names of the drugs used and their side effects has been shown to be vital in MDR TB patients recognising adverse drug reactions, seeking timely advice from healthcare workers and adhering to treatment regimens.^8^

Some respondents were incorrect in their knowledge around the duration of treatment and said they would discontinue treatment if they felt better or conversely felt worse. Although much lower in this study, the finding is supported by other studies, with a study in Tanzania reporting that up to 50% of patients with TB indicating that they would stop their treatment when ‘they feel better and think they are cured’.^18^ With treatment of MDR TB lasting 18–24 months and patients having to continue treatment until two consecutive sputum cultures are negative, the importance of continuing treatment even when patients feel better must be emphasised in patient education.

Participants in the study recognised that MDR TB was a problem in SA and that adherence to medication was essential for successful treatment of MDR TB. It has been found that patients’ attitudes can have a more profound effect on MDR TB medication adherence than knowledge.^19^ A study carried out in Eastern Cape concluded that interventions should expand beyond providing information about MDR TB and should also focus on improving attitudes and perceptions of patients and community members.^20^ This finding was about stigma against patients with MDR TB, which lead to secrecy and reluctance to visit a clinic for ongoing medication. It was further noted that a patient-centred approach is required to empower patients and that patients and their physicians should play an active role in the development and implementation of stigma reduction programmes, which deal with perceptions such as MDR TB is a punishment from God.^20^

### Limitations of the study

A limitation of the study may arise from a bias of responses in that respondents may shirk away from responding to questions that they feel are disapproved of in society – for example, responses around witchcraft. A second limitation lies in the fact that the questionnaire gathered information on patients’ reported knowledge. For example, they reported that they knew how MDR TB was diagnosed. Their actual knowledge may be incorrect and further study could assess their actual knowledge.

Information gained around attitudes from a closed-ended questionnaire may be limited as the questionnaire may not consider all potential variables around attitude. Triangulation of data could be carried out by various methods including (1) increasing the sample size, (2) repeating the study at other sites and (3) using differing data collection methods, for example, patient observation and focus group discussions.

## Conclusions and recommendations

The study of MDR TB is important because of the high incidence and prevalence in this area. In particular, a study of social aspects of MDR TB is useful. The patients attending the MDR TB Unit, which is a centralised unit, were relatively young, unemployed, with low levels of education and low income. This represents a very vulnerable population, and interventions must be sensitive to their contexts and cultures.

There is a clear need for ongoing education around aspects of the disease such as drug treatment, spread of the bacteria and duration required for treatment completion. Further research looking at the knowledge disseminated to patients by their families, friends and traditional healers may be useful. The role of traditional healers in MDR TB patient’s education requires future study. Studies on the prevalence of primary MDR TB are also necessary to confirm the high numbers found in the study and to further understand the spread of the disease and ways to reduce its transmission.

## References

[1] World Health Organization Global tuberculosis report. WHO/HTM/TB/2008.393 Geneva, Switzerland: WHO; 2012, 1–393.

[2] World Health Organization Global tuberculosis report. Geneva, Switzerland: WHO; 2014 [cited 2015 November 2]. Available from: http://www.who.int/tb/publications/global report/en

[3] Gandhi NR, Andrews JR, Brust J, et al. Risk factors for mortality among MDR and XDR TB patients in a high HIV prevalence setting. Int J Tuberc Lung Dis. 2012;16:90–97. 22236852 10.5588/ijtld.11.0153 PMC3302205

[4] Brust J, Gandhi NR, Carrara H, Osborn G, Padayatchi N High treatment failure and default rates for patients with MDR TB in KwaZulu-Natal, South Africa, 2000–2003. Int J Tuberc Lung Dis. 2010;14(4):413–419. 20202298 PMC3005763

[5] World Health Organization Multidrug and extensively drug-resistant TB (M/XDR-TB): 2010 global report on surveillance and response. WHO/HTM/TB/2010.3. Geneva, Switzerland: WHO; 2010.

[6] World Health Organization The stop TB strategy. Geneva, Switzerland: WHO; 2013 [cited 2012 Nov 2]. Available from: http://www.who.int/tb/strategy/en/

[7] Kanjee Z, Catterick K, Moll A, Amico KR, Friedland GH Tuberculosis infection control in rural South Africa: survey of knowledge, attitude and practice in hospital staff. J Hosp Infect. 2011;79(4):333–338. 21978608 10.1016/j.jhin.2011.06.017

[8] Esmael A, Ali L, Agonafir M, Desale A, Yaregal Z, Desta K Assessment of patients’ knowledge, attitude, and practice regarding pulmonary tuberculosis in eastern Amhara regional state, Ethiopia: cross-sectional study. Am J Trop Med Hyg. 2013;88(4):785–788. 23419364 10.4269/ajtmh.12-0312 PMC3617870

[9] Liam CK, Lim KH, Wong CM, Tang BG Attitudes and knowledge of newly diagnosed tuberculosis patients regarding the disease and factors affecting treatment compliance. Int J Tuberc Lung Dis. 1999;3:300–309. 10206500

[10] Buthelezi SSS Situational analysis of TB drug resistance in KwaZulu-Natal province: Republic of South Africa. 2nd Meeting of the Global XDR TB Task Force; Geneva, Switzerland; 9–10 April 2008.

[11] Johnson LF Access to antiretroviral treatment in South Africa, 2004–2011. African J of HIV Med. 2012;13(1):1–8.

[12] Jacobs TQ, Ross A Adverse effects profile of multidrug-resistant tuberculosis treatment in a South African outpatient clinic. S Afr Fam Pract. 2014;54:531–539.

[13] Westerlund EE, Marco A, Lönnermark E, Montoya R, Evans C Tuberculosis-related knowledge is associated with patient outcomes in shantytown residents; results from a cohort study, Peru. J Infect. 2015;71(3):347–357. 26033695 10.1016/j.jinf.2015.05.010 PMC7617110

[14] Sockrider MM, Wolle JM Helping patients better adhere to treatment regimen. J Respir Dis. 2005;17:204–216.

[15] Brust J, Shah NS, Scott M, et al. Integrated, home-based treatment for MDR-TB and HIV in rural South Africa: an alternate model of care. Int J Tuberc Lung Dis. 2012;16(8):998–1004. 22668560 10.5588/ijtld.11.0713 PMC3390442

[16] George LJ Compliance with medication and directly observed therapy in the treatment of TB in Lesotho. Dissertations available from ProQuest. Paper AAI3129122. 2003 [cited 2015 November 3]. Available from: http://repository.upenn.edu/dissertations/AAI3129122

[17] Norgbe GK, Smit JE, Du Toit HS Factors influencing default rates of tuberculosis patients in Ghana. Afr J Nurs Midwifery. 2011;13(2):67–76.

[18] Haasnoot PJ, Boeting TE, Kuney M Knowledge, attitudes and practice of tuberculosis among Maasai in Simanjiro District, Tanzania. Am J Trop Med Hyg. 2010;83(4):902–905. 20889888 10.4269/ajtmh.2010.10-0061 PMC2946765

[19] Cramm J, Finkenflügel F, Møller V, Nieboer A TB treatment initiation and adherence in a South African community influenced more by perceptions than by knowledge of tuberculosis. Int J Tuberc Lung Dis. 2010;14(4):413–419. 20163702 10.1186/1471-2458-10-72 PMC2828408

[20] Møller V, Erstad I Stigma associated with tuberculosis in a time of HIV/ AIDS: narratives from the Eastern Cape, South Africa. S Afr Rev Sociol. 2007;38(2):103–119.

